# Supplementary figures and images for: Mosquito bites and stage-specific antibody responses against Plasmodium falciparum in southern Ghana
Source: Malar J. 2023 Apr 15;22:126. doi: 10.1186/s12936-023-04557-8 (PMC10105943; doi:10.1186/s12936-023-04557-8)

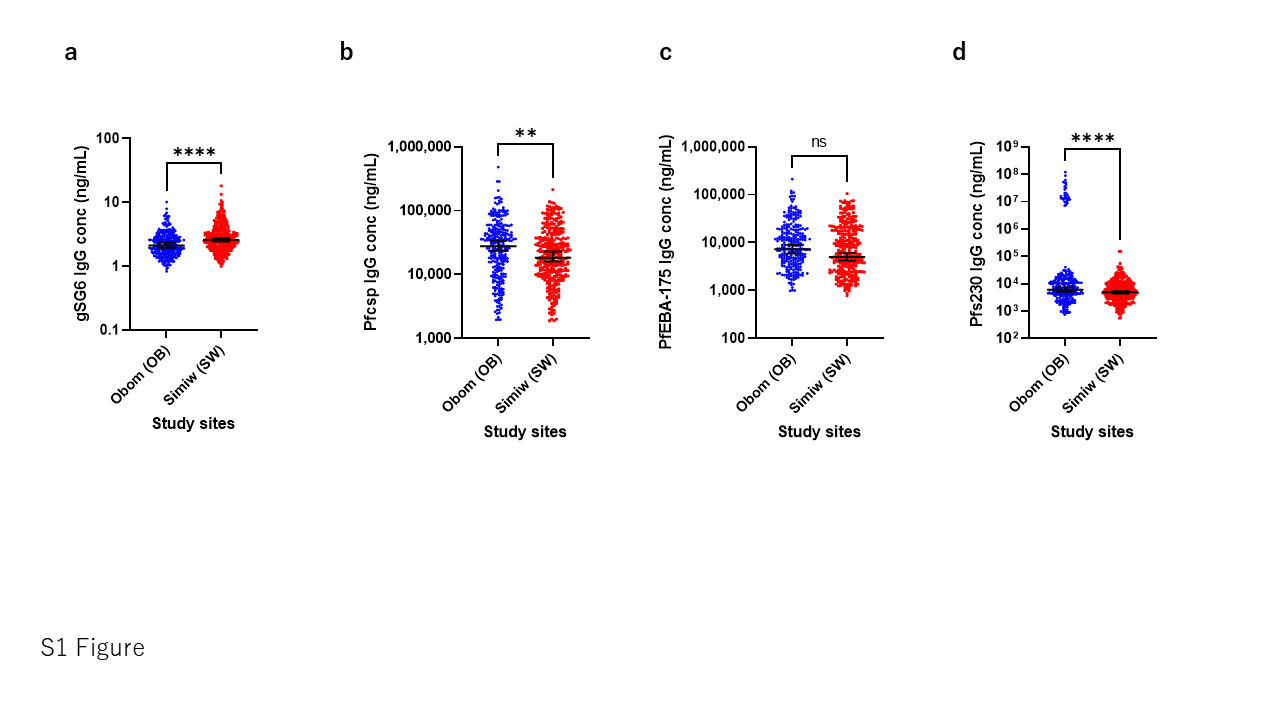

Supplement: Supplementary file 1 — Additional file 1: Figure S1. The overall Immunoglobulin G (IgG) antibody levels among the study communities. a Distribution of Anopheles salivary gland gSG6-P1; b Plasmodium falciparum CSP; c Plasmodium falciparum EBA 175; d Plasmodium falciparum Pfs230 immunoglobulin G (IgG) antibodies among the study communities. The data are represented in log10. The significance was tested using Mann Whitney U test; ns (p > 0.05) not significant; *(P < 0.05), significant; **(P < 0.01), significant; ***(p < 0.001), highly significant; ****(p < 0.0001), highly significant. [file 12936_2023_4557_MOESM1_ESM.tif]

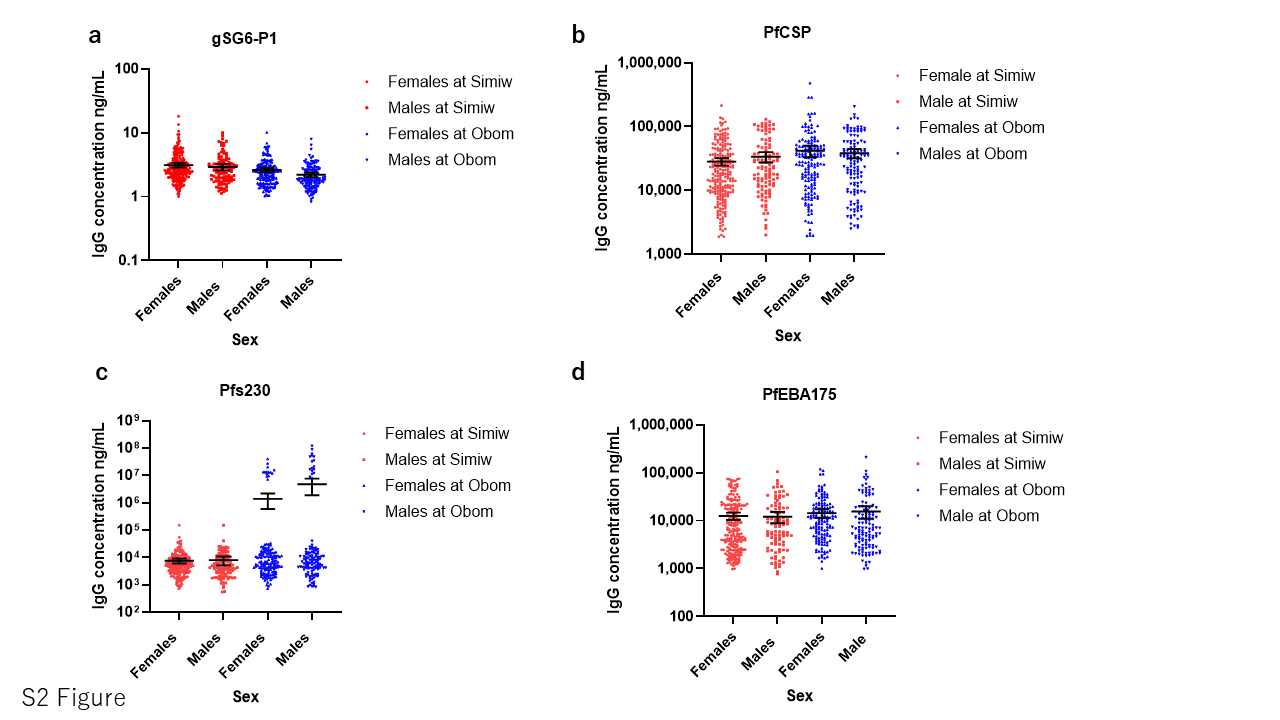

Supplement: Supplementary file 2 — Additional file 2: Figure S2. Immunoglobulin G (IgG) levels in male and female study participants. a The Anopheles salivary gland. b The P. falciparum CSP. c The P. falciparum erythrocyte binding antigen-175 (PfEBA-175). d The gametocyte surface antigen Pfs230 immunoglobulin G (IgG) antibodies concentration among male and female study participants between Obom (OB) and Simiw (SW). The data are represented in log10. [file 12936_2023_4557_MOESM2_ESM.tif]

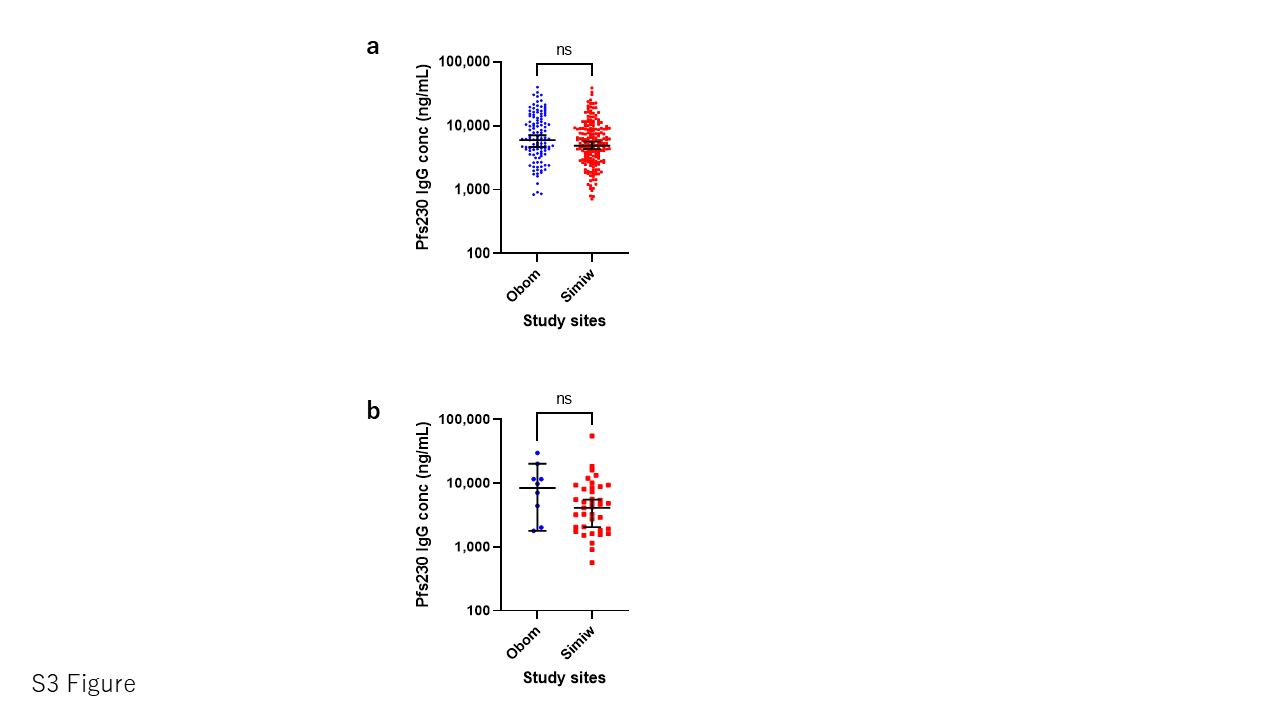

Supplement: Supplementary file 3 — Additional file 3: Figure S3. P. falciparum stage-specific IgG antibodies with similar mosquito exposures after excluding sub-groups expressing high Pfs230 IgG antibody. Association of IgG antibodies of PfCSP, Pfs230, PfEBA-175 between Obom and Simiw with a 2–5 ng/mL; b > 5 ng/mL gSG6-P1 IgG antibody exposure after excluding sub-groups expressing high Pfs230 IgG antibody at Obom. The data are represented in log10. [file 12936_2023_4557_MOESM3_ESM.tif]

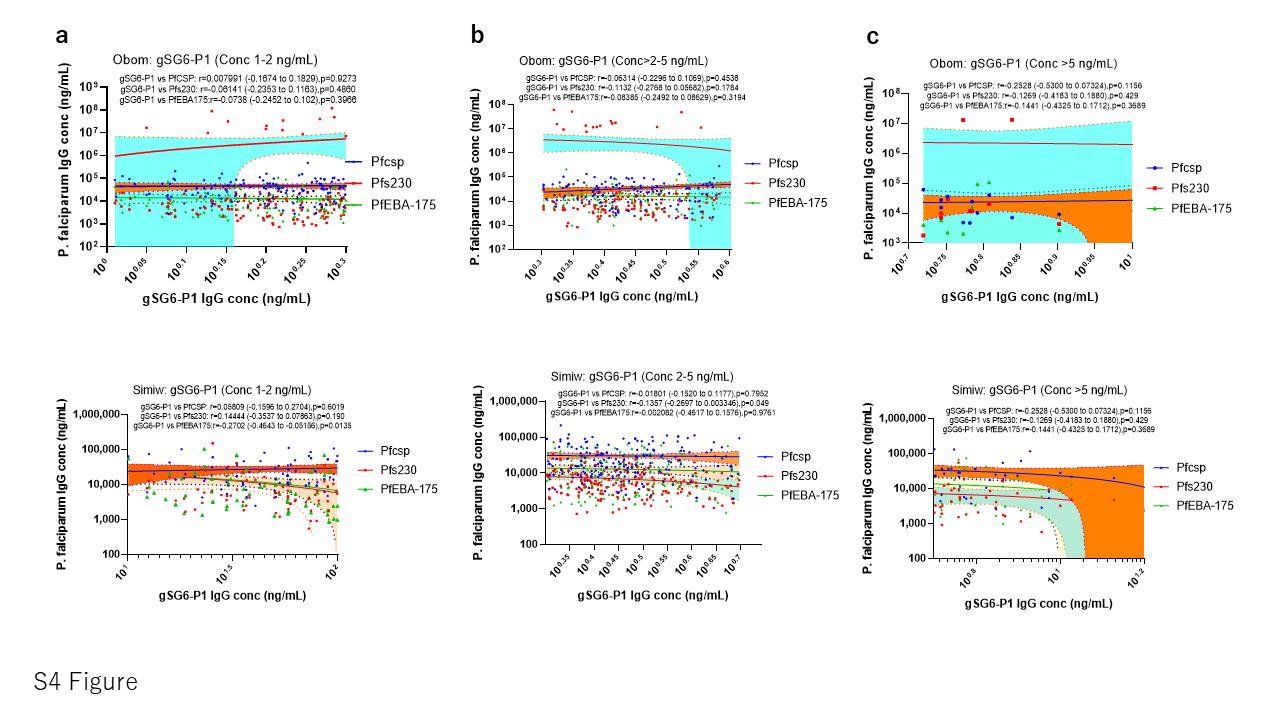

Supplement: Supplementary file 4 — Additional file 4: Figure S4. Correlation of P. falciparum stage-specific IgG antibodies across individuals with different levels of mosquito exposure. Correlation between a 1–2 ng/mL; b 2–5 ng/mL; c > 5 ng/mL gSG6-P1 IgG antibodies and PfCSP, Pfs230, and PfEBA-175 in Obom and Simiw. [file 12936_2023_4557_MOESM4_ESM.tif]
